# The malaria burden of Amerindian groups of three Venezuelan states: a descriptive study based on programmatic data

**DOI:** 10.1186/s12936-021-03819-7

**Published:** 2021-06-26

**Authors:** Juan C. Gabaldón-Figueira, Carlos Chaccour, Jorge Moreno, Maria Villegas, Leopoldo Villegas

**Affiliations:** 1 Área de Enfermedades Infecciosas, Clínica Universidad de Navarra, 31008 Pamplona, Spain; 2 Instituto de Inmunología Clínica, Universidad de Los Andes, Mérida, Venezuela; 3 ISGlobal, Hospital Clínic - Universitat de Barcelona, Barcelona, Spain; 4 Ifakara Health Institute, Ifakara, United Republic of Tanzania; 5 Centro de Investigación de Campo “Francesco Vitanza”, Instituto de Altos Estudios “Dr, Arnoldo Gabaldon”, Tumeremo, Venezuela; 6 Asociación Civil Impacto Social (ASOCIS), Tumeremo, Venezuela; 7 Global Development One, Silver Spring, MD USA

**Keywords:** Malaria, Venezuela, Amerindian groups, Amazonas, Bolivar, Sucre

## Abstract

**Background:**

Fifty-three percent of all cases of malaria in the Americas in 2019 came from Venezuela, where the epidemic is heavily focused south of the Orinoco river, and where most of the country’s Amerindian groups live. Although the disease is known to represent a significant public health problem among these populations, little epidemiological data exists on the subject. This study aims to provide information on malaria incidence, geospatial clustering, and risk factors associated to *Plasmodium falciparum* infection among these groups.

**Methods:**

This is a descriptive study based on the analysis of published and unpublished programmatic data collected by Venezuelan health authorities and non-government organizations between 2014 and 2018. The Annual Parasite Index among indigenous groups (API-i) in municipalities of three states (Amazonas, Bolivar, and Sucre) were calculated and compared using the Kruskal Wallis test, risk factors for *Plasmodium falciparum* infection were identified via binomial logistic regression and maps were constructed to identify clusters of malaria cases among indigenous patients via Moran’s I and Getis-Ord’s hot spot analysis.

**Results:**

116,097 cases of malaria in Amerindian groups were registered during the study period. An increasing trend was observed between 2014 and 2016 but reverted in 2018. Malaria incidence remains higher than in 2014 and hot spots were identified in the three states, although more importantly in the south of Bolivar. Most cases (73.3%) were caused by *Plasmodium vivax*, but the Hoti, Yanomami, and Eñepa indigenous groups presented higher odds for infection with *Plasmodium falciparum.*

**Conclusion:**

Malaria cases among Amerindian populations increased between 2014 and 2018 and seem to have a different geographic distribution than those among the general population. These findings suggest that tailored interventions will be necessary to curb the impact of malaria transmission in these groups.

**Supplementary Information:**

The online version contains supplementary material available at 10.1186/s12936-021-03819-7.

## Background

Malaria is a major public health concern in the Americas, where 889,000 cases and 550 deaths were estimated to have occurred in 2019. Fifty-three percent of these cases, and 73% of all deaths came from Venezuela, where incidence has increased over 1000% in the last two decades, amidst a context of economic crisis and political unrest [1]. The disease disproportionally affects regions south of the Orinoco River, where cases are clustered in the Sifontes municipality, of the Bolivar State [2]. There is a significantly lesser burden in the northeast coast of the country [3]. By 2016, the last year with detailed public epidemiological data, only the three states of Bolívar, Amazonas (south of the country) and Sucre (northeast) accounted for 90% of all registered cases [4]. *Plasmodium vivax* causes 77% of all registered cases, followed by *Plasmodium falciparum*, with 16%, and mixed infections (6.5%). *Plasmodium malariae* is rare and underdiagnosed [1].

The total Amerindian population in Venezuela was estimated to surpass 720,000 people in 2011 [5], most of which are concentrated in malaria-endemic regions. According to the last available census data, approximately 70% of the 76,000 Amerindians in Amazonas state lived in a rural environment. A similar picture is seen in Bolivar (79% of a 54,600 population). In Sucre, however, 60% of the 22,200 indigenous inhabitants lived in urban areas. Amerindians in Venezuela are mostly young, with a median age of 21 years, and 65% of the population are under 30 years old [5].

These indigenous groups have historically had poor access to healthcare services. In some remote areas primary healthcare and epidemiological surveillance of malaria and other infectious diseases is practiced by specially trained local community agents [6, 7]. Apart from this, these communities lack permanent access to other medical services. This, as well as the expansion of illegal mining across their territories, makes them particularly vulnerable to malaria [8, 9].

Epidemiological studies in other endemic regions of South America have demonstrated that malaria transmission in indigenous communities responds to epidemiological factors different to those in the general population [10]. Moreover, the increasingly important influence of illegal mining in said transmission dynamics has not been studied in detail and has been traditionally limited to a few settlements along the Caura River basin [7, 9, 11]. Before the COVID-19 pandemic, economic crisis had increased mobility of the general population, and likely of indigenous groups, to mines south of the country, and to Colombia, and Brazil [12]. More recently yet, restrictions caused by COVID-19 and increasingly common fuel shortages might have impeded social mobility.

Apart from a few studies evaluating the efficacy of insecticide-treated hammocks [13], and the limited distribution of bed nets in some communities [7], there are no vector control interventions that specifically target malaria transmission within indigenous communities.

This work analyses published, and unpublished epidemiological records collected by Venezuelan health authorities and non-government organizations (NGOs) between 2014 and 2018. The aim is to provide information on the general burden of malaria among the indigenous people of Amazonas, Bolivar, and Sucre states, describe differences in regional incidence, spatial clustering, risk factors for infection with *Plasmodium falciparum*, and transmission seasonality.

## Methods

This is a descriptive study based on the analysis of programmatic data collected by Venezuelan health authorities and NGOs in three Venezuelan states. Morbidity data from 2014 to 2017 was obtained from publicly available sources. These included the 2014, 2015, and 2016 Epidemiological Bulletins published by the Venezuelan Ministry of Popular Power for Health (MPPS), which present aggregated parish-level (administrative level 4) on malaria morbidity for every state [4, 14, 15], as well as the Pan-American Health Organization’s (PAHO) malaria surveillance dataset [16]. Data from 2018, as well as the ethnicity of individual cases reported during the studied period were retrieved from raw datasets and unpublished reports collected by local volunteers, and Ministry of Health’s staff (unpublished datasets, Venezuelan Ministry of Health). These datasets include names, ID numbers, age, gender, address, likely place and date of infection, place of diagnosis, identified *Plasmodium* species, occupation, and ethnicity of individual patients, and are routinely collected as part of a national passive malaria surveillance programme.

As per national guidelines, only symptomatic patients seeking care at a public health facility are tested, either with thin and thick blood films, or a rapid diagnostic test (RDT). Only new cases are included in registries, and are defined by the Venezuelan Health Ministry as any symptomatic infection with a positive test occurring 90 days or more since the completion of a treatment scheme [17]. Nonetheless, parasites are not regularly genotyped, which hampers differentiation of true new cases from relapses, given the high prevalence of * Plasmodium vivax*. Cases not meeting these criteria are considered relapses, typically removed, and stored separately by staff of the national malaria control programme.

Datasets from Amazonas, Bolivar, and Sucre were merged, and filtered to exclude patients not belonging to any of the Amerindian groups listed in the Venezuelan Ministry of Health’s registries (Additional file 1). They were then categorized based on the state of probable infection. Cases originating from states other than Amazonas, Bolivar, or Sucre were excluded from the analysis.

These data were used to estimate the risk of malaria among indigenous groups at different locations, calculating the Annual Parasite Index (API-i) at the municipality level (administrative level 3). For this, the annual cumulative malaria incidence in Amerindian patients was divided by the estimated Amerindian population of each state and municipality and multiplied by 1000. These results were compared using a Kruskal–Wallis test. The indigenous population of municipalities was estimated based on data from the last available census and using a geometric growth method, based on the following formula [18].

$$P_{n} = P_{0} \left( {1 + R_{G} } \right)^{n}$$

$$R_{G} = \frac{{\ln \left( {\frac{{P_{0} }}{{P_{{0 - 1}} }}} \right)}}{T}$$

where *P*_*n*_ represents the estimated population at any time, *n* is the time in years between the last available census and the time of estimation, *P*_*0*_ the size of the population in the last available census*, P*_*0–1*_ the population size at the second last available census, *R*_*G*_ is the estimated rate of growth and* T* represents the time between the last two available censuses*.*

Incidence trends were plotted fitting a local regression (LOESS) curve with an α smoothing factor of 0.8 into monthly time series of cases. Malaria risk for indigenous and non-indigenous inhabitants of each state and municipality was compared using relative risk (RR):

$$RR = \frac{{a/b}}{{c/d}}$$

where *a* represents malaria cases among Amerindian patients, *b* is the total estimated Amerindian population, *c* represents malaria cases among non-Amerindian patients, and *d* is the estimated non-Amerindian population. Significance was tested using a z-test.

Spatial autocorrelation of cases was determined based on the likely place of infection of individual patients, and using the global Moran’s I. Given a series of geographic entities, and an attribute associated to each one, this test evaluates if such attribute presents in a clustered, dispersed, or random pattern; with positive *I* values indicating clustering. This test, however, fails to pinpoint individual clusters, and is of limited value when the spatial pattern is not homogenously distributed [19]. Thus, individual clusters of high (hot spots) and low (cold spots) infection burden were also mapped applying the Getis-Ord’s Gi* statistic to reported parishes of infection, except in Amazonas, where it was applied to municipalities, due to the lack of adequate parish-level data. This test compares the mean number of local cases in a geographical unit and its neighbours, to the global mean, assigning a *p* and *z* value to each one. Large positive *z* values and small *p* values indicate a significant hot spot, while large negative *z* values and small *p* values suggest a significant cold spot [20].

The significance of clustering was determined with a *z*-score and parishes were classified based on it: confidence 90% (0.1 > *p* > 0.05), confidence 95% (0.05 > *p* > 0.01) and confidence 99% (*p* < 0.01), as done in similar studies [20, 21].

The proportion of malaria cases caused by different *Plasmodium* species was compared using the Chi-square test. Odds ratios in univariate and multivariable models for *P. falciparum* vs *P. vivax* infection were calculated via binomial logistic regression and *p* values determined via Wald’s test.

Data analysis and processing was performed using Excel 2016 (Microsoft, Richmond, Virginia), SPSS 25 (IBM, Armonk, New York) and RStudio V 1.3.1093 (RStudio Team, Boston, Massachusetts). ArcGIS Pro 24 (Esri, Redlands, California) was used to construct maps and perform spatial analysis. Significance was set at 0.05.

## Results

### Malaria incidence among indigenous communities in Amazonas, Bolivar and Sucre

**Fig. 1:**
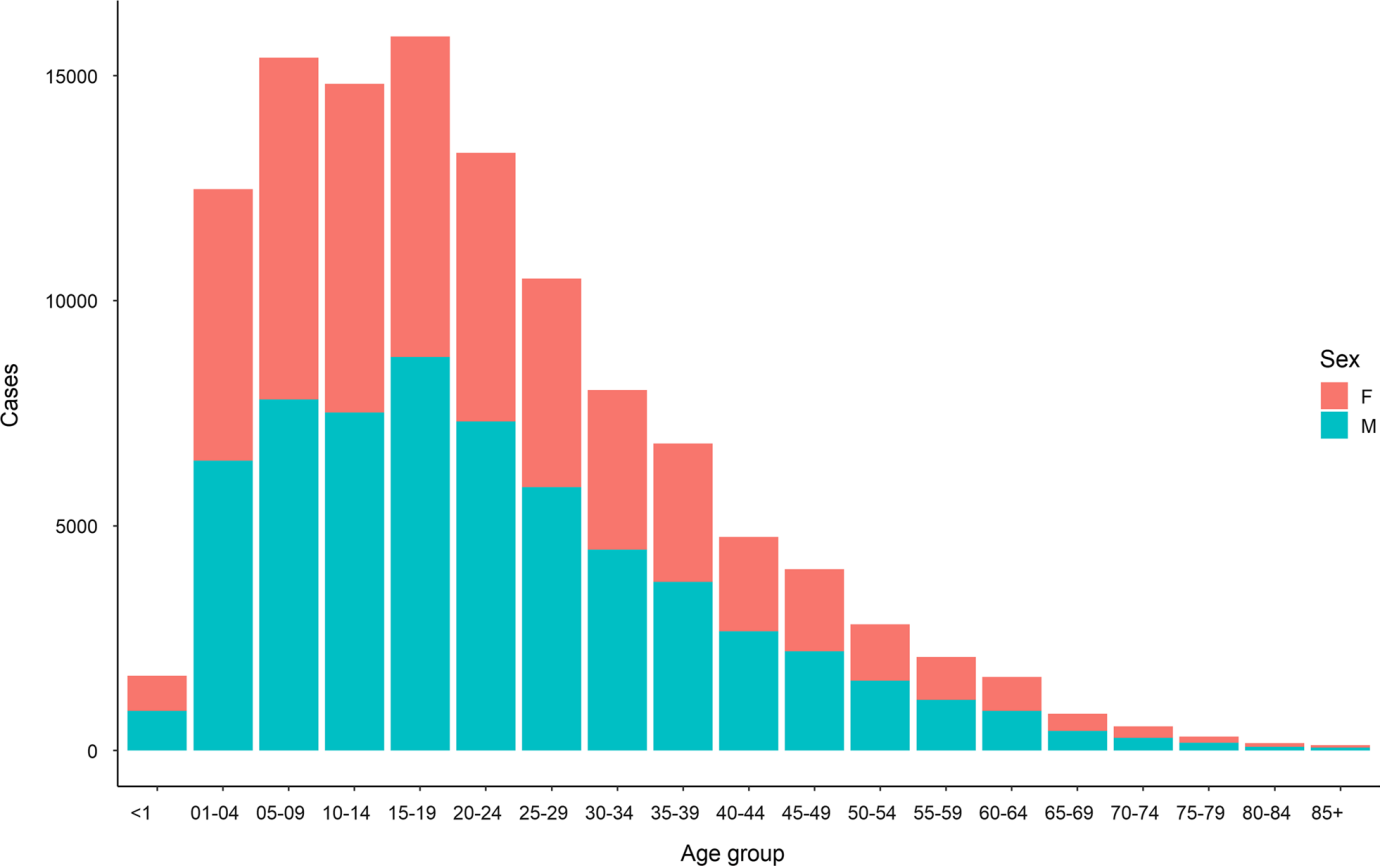
Age and sex distribution of malaria cases registered in Amerindian groups of Amazonas, Bolivar, and Sucre, 2014–2018

A total of 116,097 new cases of malaria were registered in the 2014–2018 period among Amerindians. Of these, 62,267 (53.6%) were male and 53,830 (46.4%) female. Median age was 19 years (IQR: 9–31 years, Fig. 1), and 85% of all patients were under 40 years old.

Studied patients belonged to 39 different ethnic groups. However, only nine accounted for 91% of cases. These are: Pemon (27.3%), Jivi (25%), Piaroa/Wotjuja (16.1%), Yekuana (8.9%), Kurripaco (3.9%), Eñepa (2.9%), Piapoko (2.9%), Yanomami (2.8%) and Baré (1.3%). Demographic characteristics of individual Amerindian groups are summarized in Table 1.

**Table 1:** Distribution of cases of malaria among Amerindian groups by sex, origin of infection, and ethnicity, 2014–2018

Group	Female	Male	Am	Bol	Su	Total	(%)	Median age	IQR
Akawayo	215	748	2	961	0	963	0.8	28	13
Amorua	1	0	0	0	1	1	0	29	(NA)
Añu	0	1	0	1	0	1	0	11	(NA)
Arawak	12	37	3	45	1	49	0	27	30
Baniva	487	523	997	13	0	1010	0.9	31	28
Baré	632	857	1464	25	0	1489	1.3	30	27
Barí	1	0	1	0	0	1	0	31	(NA)
Chirichano	2	0	0	2	0	2	0	22	5
Eñepa	1625	1702	16	3310	1	3327	2.9	15	22
Guanono	34	23	56	1	0	57	0	17	20
Hoti	501	441	443	498	1	942	0.8	9	18
Inga	26	30	17	39	0	56	0	29	24
Japreria	1	1	1	1	0	2	0	19	5
Jivi	14,219	14,773	26,112	2880	0	28,992	25	17	21
Kariña	742	724	0	1459	7	1466	1.3	14	19
Kubeo	13	16	25	4	0	29	0	20	24
Kuiva	1	2	3	0	0	3	0	53	37
Kurripaco	2152	2418	4449	121	0	4570	3.9	23	24
Mako	489	556	1043	2	0	1045	0.9	17	19
Panare	27	30	57	0	0	57	0	15	25
Pemon	13,119	18,571	4	31,685	1	31,690	27.3	20	21
Piapoko	1582	1730	2313	999	0	3312	2.9	16	18
Piaroa/Wotjuja	9019	9691	16,424	2286	0	18,710	16.1	20	21
Puinave	560	616	1139	16	21	1176	1	21	25
Pume	1	4	4	1	0	5	0	37	39
Saliva	236	293	513	16	0	529	0.5	21	23
Sanema	541	592	93	1040	0	1133	1	14	19
Sape	1	1	2	0	0	2	0	18	15
Timotocuica	1	1	1	1	0	2	0	22	42
Uruak	14	16	1	0	29	30	0	17	24
Wanai	91	85	64	112	0	176	0.1	27	25
Warao	193	211	1	175	228	404	0.3	15	21
Warekena	173	144	315	1	1	317	0.3	23	24
Wayuu	10	19	12	17	0	29	0	22	25
Yanomami	1621	1633	3199	55	0	3254	2.8	9	15
Yebarana	142	118	259	1	0	260	0.2	20	22
Yekuana	5037	5323	6001	4359	0	10,360	8.9	19	21
Yeral	306	337	640	3	0	643	0.6	27	25
Yukpa	3	0	1	2	0	3	0	18	39
Total	53,830	62,267	65,675	50,131	291	116,097	100	19	22

*Am* Amazonas, *Bol* Bolivar, *Su* Sucre, *IQR* Interquartile range

Most cases were registered in Amazonas (56.6%) followed by Bolivar (43.2%) and Sucre (0.25%). The year with the highest number of new malaria cases was 2017 with 30,976.

#### Amazonas state

**Fig. 2:**
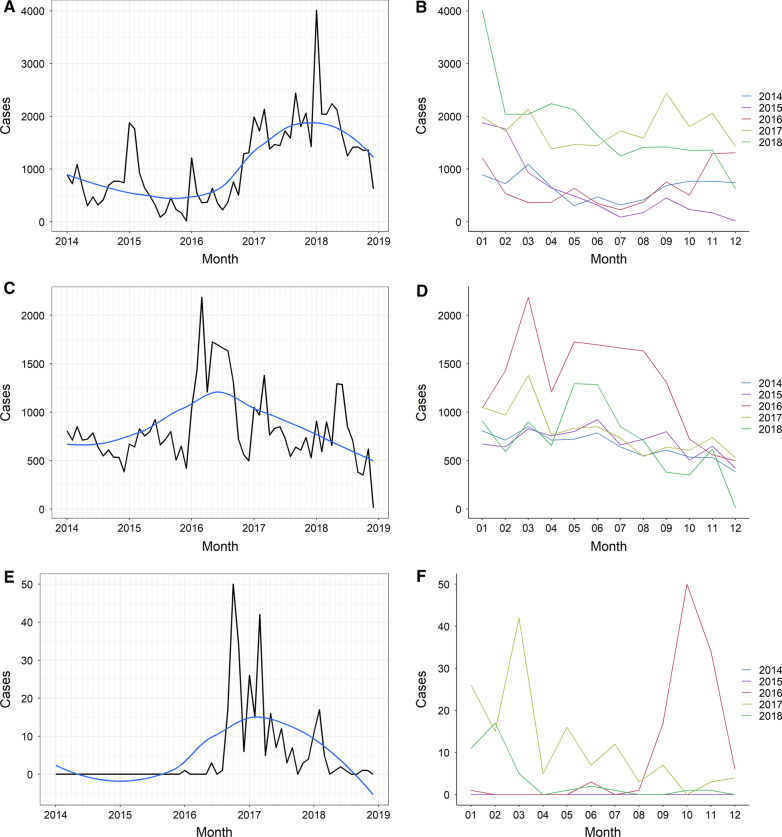
Monthly malaria cases registered in Amerindian groups of Amazonas, Bolivar and Sucre states, Venezuela, 2014–2018. Monthly cases registered between 2014 and 2018 in Amazonas (**A**), Bolívar (**C**) and Sucre (**E**), and aggregated cases per month and year in the three states (**B**, **D**, and **F**, respectively). The blue line in the panels on the left represents the LOESS curve

Most patients belonged to ethnic groups located in the north of the state, namely, Jivi (39.8%), Wotjuja (25%) and Yekuana (9.1%). The number of new malaria cases in indigenous communities in 2018 (21,530), was 174.2% higher than compared to the 2014 baseline (7852). An increasing trend in the number of monthly cases was observed between January 2016 and January 2018, when cases started to decrease (Fig. 2A). Despite this, the relative malaria risk for indigenous people compared to the non-indigenous population fell from 2.44 in 2014 (*p* < 0.01), to 0.89 (*p* < 0.01) in 2018 (Table 2). Cases in Amazonas were typically higher between January and June, but a clear seasonal pattern was not observed (Fig. 1B), and no significant differences were seen in the median number of monthly cases during the study period (*p* = 0.57, Additional file 5).

**Table 2:** Cumulative incidence of malaria among indigenous and non-indigenous groups in Amazonas, Bolivar and Sucre states, Venezuela, 2014–2018

Year	Cases-i	Est. Pop-i	API-i	Cases non-i	Est. Pop non-i	API non-i	RR
Amazonas							
2014	7852	82,800	94.83	3512	90,344	38.87	*2.44
2015	7164	85,082	84.20	11,477	92,921	123.51	*0.68
2016	7942	87,427	90.84	17,009	95,481	178.14	*0.51
2017	21,187	89,837	235.84	45,113	98,015	460.27	*0.51
2018	21,530	92,313	233.23	26,283	100,522	261.47	*0.89
Bolivar							
2014	7854	57,451	136.71	67,089	1,665,910	40.27	*3.39
2015	8387	58,404	143.60	98,466	1,693,846	58.13	*2.47
2016	15,676	59,372	264.03	161,943	1,721,527	94.07	*2.81
2017	9649	60,357	159.87	251,673	1,748,949	143.90	*1.11
2018	8565	61,357	139.59	242,801	1,776,128	136.70	*1.02
Sucre							
2014	0	23,256	0.00	922	988,415	0.93	0
2015	0	23,615	0.00	3208	1,003,939	3.20	0
2016	112	23,978	4.67	20,821	1,019,515	20.42	*0.23
2017	140	24,348	5.75	61,747	1,035,140	59.65	*0.10
2018	39	24,723	1.58	67,992	1,050,722	64.70	*0.02

Spatial autocorrelation and clustering

*Est. Pop* Estimated population, *API* Annual parasite index, *i* Indigenous people, *Non-i* Non-Indigenous people (cases per 1000 people at risk), *RR* Risk ratio indigenous/non-indigenous

Z test for significance: *p < 0.05

**Fig. 3:**
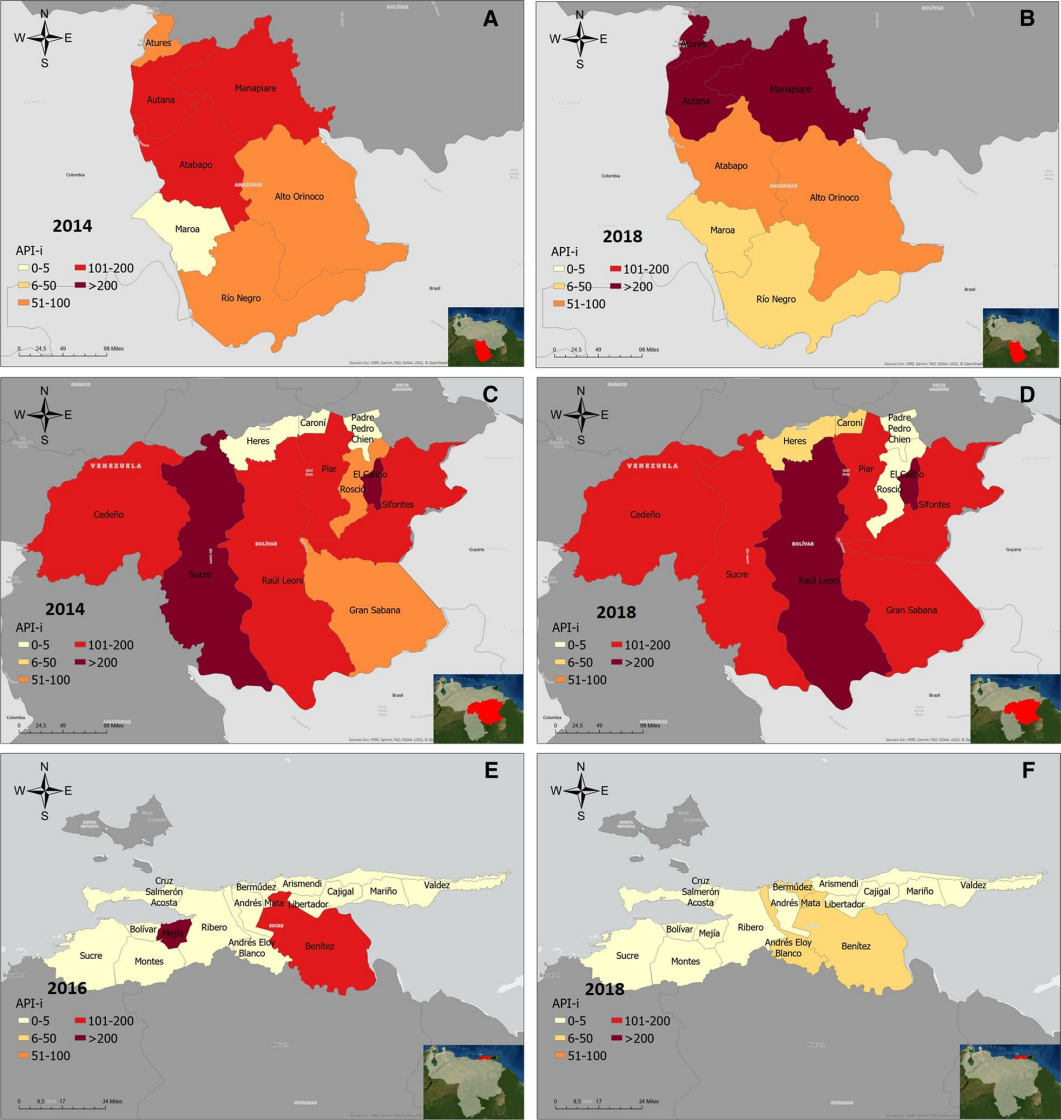
Evolution of malaria incidence in Amerindian groups of Amazonas, Bolivar and Sucre states, Venezuela,  2014 and 2018. Annual Parasite Index among indigenous patients (API-i) in Amazonas (**A**, **B**), Bolivar (**C**, **D**), and Sucre (**E**, **F**). Panels on the left represent 2014 data (except Sucre), panels on the right, 2018

At municipal level, the Atures municipality accounted for most cases during the five-year study period (55.28%). There was a significant difference in the median incidence of municipalities (*p* < 0.01), with the highest median API-i registered in Manapiare: 249.05 (IQR:151.25–301.64, Fig. 3, Additional file 2).

#### Bolivar state

The Pemon, the most numerous ethnic group in the state, accounted for 63.2% of all cases in Bolivar, followed by the Yekuana (8.7%) and the Eñepa (6.6%). As in Amazonas, malaria cases increased during the study period, compared to the 2014 baseline (7854), but particularly in 2016 (15,676). From this point on, incidence reduced, reaching 8565 cases in 2018, 9.05% more than in 2014 (Fig. 2C). Compared to that in the rest of the population, malaria risk for indigenous people was consistently higher in Bolivar: RR: 3.39 in 2014 (*p* < 0.01), 2.47 in 2015 (*p* < 0.01), 2.80 in 2016 (*p* < 0.01), 1.11 in 2017 (*p* < 0.01) and 1.02 in 2018 (*p* = 0.04, Table 2). A seasonal pattern seems more patent in this state, as cases peak between February and April, and start declining from June onwards (Fig. 2C, D). There was a significant difference in the median number of monthly cases throughout the five-year period (*p* < 0.01).

At municipal level, almost a quarter of all cases originated in the Gran Sabana municipality (24.2%), followed by Angostura (formerly known and noted in maps as Raul Leoni, 23.3%) and Cedeño (20.4%) municipalities. Incidence in the study period was significantly different between municipalities (*p* = 0.012), with the highest median API-i registered in El Callao: 575.76 cases per 1000 indigenous people (IQR: 457.14–1205.88). This, however, is probably a result of the extremely small projected indigenous population in the municipality (Additional file 3). Angostura had the highest median API-i of all municipalities with 100 indigenous inhabitants or more (310.84, IQR: 207.81–334.57, Fig. 3, Additional file 2).

#### Sucre state

The Warao accounted for 228 cases (78.4%), followed by the Uruak (10%), and the Puinave (7.2%). Although the number of cases remained considerably lower than in Amazonas and Bolivar, it increased from zero in 2014 and 2015 to 140 in 2017, followed by a 72% reduction in 2018 (39 cases). Annual incidence peaked in 2017 (5.75 cases per 1000 indigenous people), and then reduced to 1.58 in 2018 (Fig. 2E, Table 2).

At municipal level, Benítez registered the highest API-i of the state (174.52, IQR: 0–174.52), however, this difference was not significant (*p* = 0.06) when municipalities with an indigenous population below 100 were excluded from the analysis. There is no evident seasonal pattern in Sucre (Fig. 2F).

The origin (place of likely infection) of malaria cases registered in 2014 (2016 for Sucre) and 2018 was mapped to compare changes in geographic clustering. Maps were constructed to the parish level (fourth administrative level) in Bolivar and Sucre, and to the municipality level (third administrative level) in Amazonas, due to the lack of adequate parish-level data in this state.

**Fig. 4:**
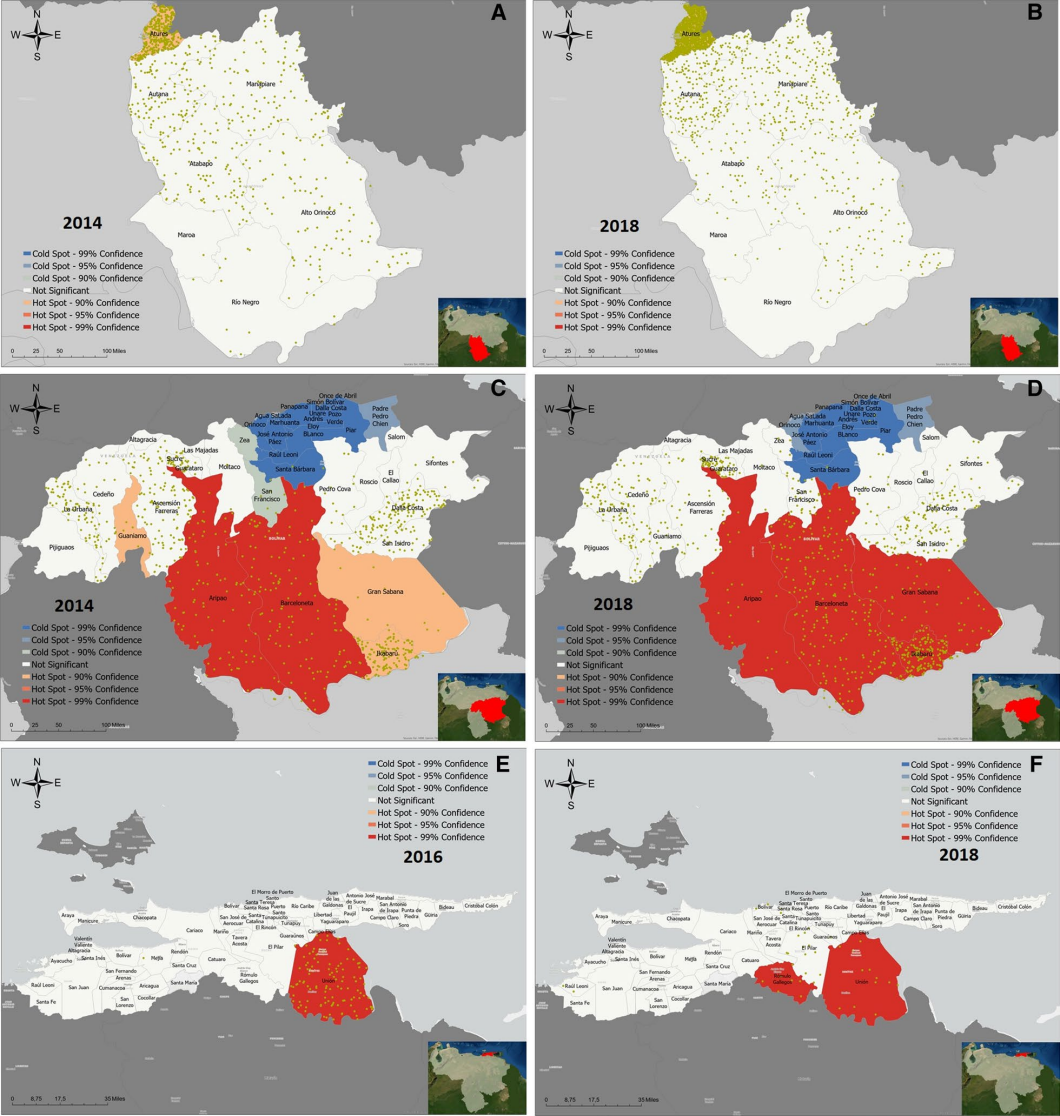
Evolution of clustering of malaria cases diagnosed among indigenous groups of Amazonas, Bolivar, and Sucre states, Venezuela, 2014–2018. Burden hot and cold spots detected in Amazonas (**A**, **B**), Bolivar (**C**, **D**) and Sucre (**E**, **F**). Panels on the left represent 2014 data, panels on the right, 2018. Data of 2016 is not shown, except for Sucre, where no cases were registered in 2014. Each dot represents 10 cases of malaria in indigenous people (Amazonas and Bolivar) or 1 case (Sucre). Confidence: 99% (p < 0.01), 95% (0.05 > p > 0.01), 90% (0.1 > p > 0.05)

The Atures municipality accounted for most cases in Amazonas (44.1% in 2014, and 67.5% in 2018). Yet, Moran’s I showed no significant clustering either year. The Getis-Ord analysis, however, revealed a low-significance hot spot (confidence 90%) in Atures (Fig. 4A, B).

In Bolivar, the Moran’s I was positive and statistically significant in 2014 (*I* = 0.19, *p* = 0.006) and 2018 (*I* = 0.17, *p* = 0.006) indicating stable clustering of cases. This was reflected in the hot spot analysis: In 2014, two highly significant (confidence 99%) hot spots were seen in Aripao (Sucre municipality) and Barceloneta (Angostura municipality). Hot spots of lower significance (confidence 90%) were seen in Guaniamo (Cedeño), Gran Sabana and Ikabaru (Gran Sabana municipality, Fig. 4C, D). New high-significance clusters appeared in the entire Gran Sabana municipality in 2018. The central northern region of the state remained a high-significance cold spot during the entire study period.

The situation in Sucre state was only compared to 2016, due to the absence of cases in the two previous years. No significant clusters were identified via Moran’s I analysis. The Getis-Ord’s Gi* revealed highly significant hot spots in Union in 2016 and 2018 (Benitez municipality), and Romulo Gallegos (Andrés Eloy Blanco municipality) in 2018.

The full results of the Getis-Ord’s analysis can be seen in Additional file 4.

### Proportion of *Plasmodium* species and risk factors associated to *Plasmodium falciparum* infection

Most infections were caused by *Plasmodium vivax*: 85,124 (73.3%), * Plasmodium falciparum*: 25,201 (21.7%), or both: 5726 (4.9%)*. Plasmodium malariae* was only identified in 46 patients (0.04%), from eight ethnic groups, all of them from Amazonas, and mostly infected in Alto Orinoco (31 cases), and Manapiare (8 cases). The Yanomami accounted for 58.7% of all *Plasmodium. malariae* cases. While * Plasmodium vivax* was the dominant species in all the states, the proportion of *Plasmodium falciparum* cases in Amazonas and Bolivar (22% and 21.4%, respectively) was more than twice that in Sucre state (8.6%, X^2^ = 35.82 df = 2, *p* < 0.01).

The proportion of patients with *Plasmodium falciparum* or mixed infection was also higher in two ethnic groups: the Hoti (42.9%) and the Yanomami (41.5.%), compared to the rest of the indigenous population (26.1%, X^2^ = 128.30, df = 1, *p* < 0.01 and X^2^ = 380.83, df = 1, *p* < 0.01, respectively).

To evaluate possible reasons behind this difference, the odds ratios (OR) for *Plasmodium falciparum* vs. *Plasmodium vivax* infection were calculated considering ethnic group, and other available variables such as occupation, gender, age group, and state of infection, results are summarized in Table 3. *Plasmodium malariae* and mixed infections were excluded from the analysis.

**Table 3:** Univariate and multivariable analysis of the odds ratios for *Plasmodium falciparum* infection among indigenous groups of Amazonas, Bolivar and Sucre states, Venezuela, 2014–2018

Occupation	*Pv*	*Pf*	Univariate				Multivariable			
			OR (Pf/Pv)	CI		*p* value	OR (Pf/Pv)	CI		*p* value
Mining	7763	2656	1.44	1.35	1.54	< 0.01	1.09	1.01	1.18	0.03
Agriculture	2951	886	1.26	1.15	1.39	< 0.01	0.82	0.74	0.91	< 0.01
Commerce	8371	2873	1.45	1.35	1.55	< 0.01				
Student	8391	2257	1.13	1.06	1.21	< 0.01				
Others	7268	1726	1.00							
Age group										
5–14	23,101	5619	1.02	0.97	1.07	0.50	1.08	1.02	1.15	0.01
15–29	28,739	8893	1.30	1.23	1.36	< 0.01	1.47	1.39	1.56	< 0.01
30–64	21,127	7565	1.50	1.43	1.58	< 0.01	1.75	1.64	1.85	< 0.01
65+	1307	550	1.76	1.58	1.96	< 0.01	2.07	1.85	2.32	< 0.01
0–4	10,424	2489	1.00							
Gender										
Female	39,443	11,876	1.03	1.00	1.06	0.03	1.04	1.01	1.08	0.01
Male	45,681	13,325	1.00							
Ethnic group										
Pemon	22,002	6633	1.19	1.12	1.26	< 0.01	1.17	1.09	1.25	< 0.01
Jivi	21,332	6701	1.24	1.17	1.32	< 0.01	1.29	1.22	1.37	< 0.01
Yekuana	7804	2328	1.18	1.10	1.26	< 0.01	1.21	1.13	1.30	< 0.01
Wotjuja	14,640	3429	0.93	0.87	0.99	0.02				
Kurripaco	3591	812	0.89	0.82	0.98	0.02	0.87	0.79	0.96	< 0.01
Eñepa	2335	946	1.60	1.46	1.75	< 0.01	1.76	1.59	1.94	< 0.01
Piapoko	2500	712	1.13	1.02	1.24	0.02	1.20	1.09	1.33	< 0.01
Yanomami	1875	1248	2.63	2.41	2.87	< 0.01	3.08	2.81	3.38	< 0.01
Hoti	538	389	2.86	2.48	3.29	< 0.01	3.33	2.89	3.84	< 0.01
Kubeo	25	3	0.47	0.14	1.57	0.22				
Warao	325	48	0.58	0.43	0.79	< 0.01				
Warekena	255	44	0.68	0.49	0.94	0.02	0.67	0.48	0.93	0.02
Akawayo	791	108	0.54	0.44	0.66	< 0.01	0.46	0.38	0.57	< 0.01
Other	7111	1800	1.00							
State										
Amazonas	48,894	14,452	2.92	1.93	4.41	< 0.01	2.52	1.49	4.29	< 0.01
Bolivar	35,983	10,724	2.94	1.95	4.45	< 0.01	2.31	1.38	3.87	< 0.01
Sucre	247	25	1.00							

*Pf*, *Plasmodium falciparum*; *Pv*, *Plasmodium vivax*; OR, Odd ratios calculated via binomial logistic regression; CI, 95% confidence intervals; p values calculated via Wald’s test

Data on occupation was only recorded in 40.9% of the 110,325 patients with either *Plasmodium falciparum,* or *Plasmodium vivax* infection, most of which worked in the commercial and services branch of the economy (10.3%), followed by students (9.9%), miners (9.8%), and those working in agriculture (3.4%). The remaining 8.2% of all patients with information on occupation worked in diverse areas that were included in the group “others”. Compared to this group, miners had a small, but significantly higher OR for *Plasmodium falciparum* infection (1.09, CI 1.01–1.18, *p* = 0.03).

Odds ratios for *Plasmodium falciparum* infection increased significantly with age. Compared to children under 5 years, patients over 65 had the highest ratio (OR: 2.08, CI 1.85–2.33, *p* < 0.01). Similarly, odds ratios were also considerably higher for the Hoti (OR: 3.33, CI 2.89–3.84, *p* < 0.01), the Yanomami (OR: 3.08, CI 2.81–3.38, *p* < *0.01*), and to a lesser extent, the Eñepa (1.76, CI 1.59–1.94, *p* < 0.01) as well as for patients from Amazonas (OR: 2.52. CI 1.49–4.29, *p* < 0.01), and Bolivar (OR: 2.31, CI 1.38–3.87, *p* < 0.01).

## Discussion

Malaria seems to affect Amerindian patients similarly regardless of sex, with most cases concentrating in patients between 5 and 24 years (50.3%). Symptomatic disease reduces in older groups, as the proportion of cases in male patients increases, likely due to a higher exposure to infectious bites.

The number of cases of malaria among Amerindian patients per 1000 people at risk (API-i) increased between 2014 and 2018 in all the studied states, mainly driven by a surge in cases in Amazonas, and to a lesser extent in Bolivar, which was particularly clear between 2014 and 2017. From that point onwards, cases in these two states have stabilized, and even returned to levels similar to those of 2014 in the case of Bolivar. This probably responds to recent efforts carried out by local and international actors, which have concentrated on this State.

Furthermore, compared to that of the non-indigenous population, malaria risk among Amerindian groups has reduced since 2014, indicating that the current national epidemic is mostly driven by non-indigenous patients.

There are obvious differences in risk between different municipalities, with API-is in Manapiare (Amazonas), El Callao, and Angostura (Bolivar) being particularly high. However, the municipal API-i should be interpreted carefully, as the malaria risk of communities with a small projected indigenous population is likely overestimated. Population estimates used in this study are entirely based on census data, which does not include transient inhabitants who are living and working in the study area. This is a major limitation of this study, as the actual at-risk population in these places is certainly larger than estimated. Places like El Callao (Bolívar), where registered malaria cases exceed the projected indigenous population, leading to abnormally large API-is, are a good example of this. A full list of the estimated indigenous populations of individual municipalities can be found in the Additional file 3. To address this issue and prioritize areas for eventual interventions, the cluster analysis was carried out with individual cases, rather than calculated API-is.

In Amazonas state, although most cases originated in Atures (where the only large town in the state is located), the Getis-Ord’s Gi* statistic only revealed low-significance hotspots both in 2014 and 2018. This suggests that cases are only weakly clustered, and more distributed throughout the north of the state (Fig. 4). Although the lack of parish-level data in Amazonas limits the interpretation of these results, this might be partially influenced by the Orinoco Mining Arc, a large-scale mining project involving areas along the border between Bolivar and Amazonas [22, 23]. Increased domestic migration from other states due to mining might also help explain the reduction in the relative risk of Amerindian groups, compared to non-indigenous patients in this state.

In Bolivar State, clustering was consistently identified south of the state. The southeast of Bolivar comprises the historical location of the Pemon, the largest single ethnic group identified in this study. Illegal mining by this and other groups in protected areas of Canaima National Park [24], mostly located in the Gran Sabana municipality, and along the Caroní River (the natural border between Barceloneta and Gran Sabana parishes), is well reported [22, 24, 25]. Smaller-scale illegal mining in the remote Upper Caura river basin (Aripao parish), and the Paragua river (Barceloneta parish), mostly conducted by local Amerindian groups like the Yekuana, has also been documented [25–27], and likely influences the clustering of malaria among Amerindian communities in the south of Bolivar. The highly significant cold spots identified in the north of the state respond to the small proportion of indigenous population in the area.

Surprisingly, the Sifontes municipality, recently identified as the most important cluster of malaria transmission in the Americas [2], was not found to be a significant hot spot for Amerindian groups. While this might reflect a predominance of mining activity by the Pemon, the Yekuana, and other groups in different areas, further research is needed to confirm this finding and understand the reasons behind it.

If malaria among Amerindian groups indeed presents a different geographic pattern, interventions in locations apart from Sifontes will be key to curb the impact of the epidemic. Geolocation data used in this work comes from interrogation of confirmed cases by Ministry of Health staff. Therefore, although this data can provide a general picture of the spatial distribution of infections, global positioning system (GPS) data should be obtained from active case detection campaigns carried out in parishes identified as hot spots, to objectively confirm these findings, and guide future interventions.

Cases in Sucre represented only 0.25% of all cases analysed, in accordance with its comparatively small and mostly urban indigenous population, and less intense transmission. Hot spots in this state matched areas of known high incidence [16], where indigenous patients have probably benefited more from interventions currently in place, explaining the reduction seen in 2018.

All data used in this study came from passive detection of symptomatic malaria cases. Therefore, annual cumulative incidence is likely underreported, as some patients will develop asymptomatic infection, and many more will not seek medical attention at all. The effect of this underreporting heterogeneously impacts the interpretation of these results, as it is probably more important among isolated groups, (such as the Yanomami, the Hoti, or the Yekuana) than others that are better connected to the national health infrastructure (Pemon) or live in a predominantly urban environment (patients infected in the north of Bolivar, and those living in Sucre state). Similarly, cases originated in these states, but reported elsewhere, were not included in the analysis.

As only new cases meeting the Ministry of Health’s definition are included in the datasets, duplicated cases are generally excluded and stored separately by Ministry staff, which reduces the potential impact of misreporting.

Seasonality was only observed in Bolivar, but this has not been described in the general population [28]. However, these studies date from 2010, and malaria cases in this state are influenced by the El Niño Southern Oscillation phenomenon [28], which was particularly intense in the 2015–2016 period [29] and might have influenced the large peak of cases in the early months of 2016 and 2017. The exposure of the indigenous population to infectious bites might also change more markedly during the year due to specific cultural or economic factors beyond the scope of this study.

The presence of *Plasmodium malariae* exclusively in patients from Amazonas, and mostly among the Yanomami, matches previous reports that link this species to remote areas of the state [30]. The Hoti and the Yanomami were also found to have significantly higher odds for *Plasmodium falciparum* infection. Similar to *Plasmodium malariae, Plasmodium falciparum* is more prevalent in the rainforest of Amazonas than in the rest of the country [31]. Previous works suggest that the higher prevalence of *Plasmodium falciparum* and *Plasmodium malariae* in the Alto Orinoco region might be explained by a longer life expectancy of local *Anopheles darlingi* mosquito populations [31]. This might in turn reflect flaws in local vector control strategies, and the lack of targeted measures that adapt to the particular living conditions of the Yanomami and the Hoti, such as their nomadic habits, and housing materials that render IRS and bed nets ineffective [31]. Insecticide-treated hammocks have proven a useful alternative in this context [13].

Although miners presented higher odds for *Plasmodium falciparum* infection too, the difference was very small and might be more related to their increased exposure to infectious bites, than to a higher *Plasmodium falciparum* prevalence in Venezuelan mines, which has not been described [12]. Furthermore, occupation data was only available for 45,142 patients (40.9% of the total analysed), complicating the interpretation of these results. Increased exposure to infectious bites is probably also the reason for higher odds in older age groups.

## Conclusion

Malaria incidence among Amerindian groups has increased since 2014, although the trend has partly reversed in the last two years. Clustering of malaria cases is particularly clear in Bolivar, where it is likely being influenced by mining, necessitating a wider deployment of targeted interventions. These interventions should initially prioritize the Hoti, Yanomami, and Eñepa, due to their increased risk of *P. falciparum* infection, associated with severe malaria.

## Supplementary Information

**Additional file 1.** Ethnic groups included in registries of the Venezuelan Ministry of Health (MPPS).

**Additional file 2.** Malaria incidence in indigenous patients and relative risk compared to non-indigenous population.

**Additional file 3. **Estimated indigenous population and growth rates.

**Additional file 4. **Getis-Ord’s Gi* Analysis for clustering of malaria cases (2014–2018).

**Additional file 5. **Seasonal distribution of malaria cases diagnosed among Amerindian patients in Amazonas, Bolivar and Sucre between 2014 and 2018.

## Data Availability

The datasets used for this project are available from the corresponding author on reasonable request.

## Abbreviations

API-i: Annual Parasite index among Amerindian groups (number of cases of malaria reported in Amerindian patients per 1000 Amerindian people)
GPS: Global positioning system
NGO: Non-government organization
