# Supplementary material for: The malaria burden of Amerindian groups of three Venezuelan states: a descriptive study based on programmatic data
Source: Malar J. 2021 Jun 26;20:285. doi: 10.1186/s12936-021-03819-7 (PMC8235908; doi:10.1186/s12936-021-03819-7)
Supplement: Supplementary file 1 — Additional file 1. Ethnic groups included in registries of the Venezuelan Ministry of Health (MPPS). [file 12936_2021_3819_MOESM1_ESM.docx]

**Ethnic groups included in registries of the Venezuelan Ministry of Health (MPPS)**

- Akawayo (Akawaio, Kapon)
- Amorua
- Añu
- Arawak (Arauko, Arawko Lokono)
- Ayaman
- Baniva
- Baré
- Barí
- Chaima.
- Cubeo
- Cumanagoto
- E´ñepá (Panare)
- Guanono
- Hoti ( Jodi, Hodi, Joti)
- Inga
- Japrería
- Jivi (Jiwi, Guahibo, Sikuani)
- Kariña
- Kuiva (Cuiva, Cuiba)
- Kurripaco (Curripaco, Baniwa, Wakuénai)
- Mako
- Ñegantú (Yeral)
- Pemón (Taurepan, Arekuna)
- Piapoko (Tsaase)
- Puinave
- Pumé (Yaruro, capuruchano)
- Sáliva
- Sanema (Sanima, Sanuma, Guaica)
- Sape
- Timoto-Cuicas (Timotes)
- Uruak (Arutani)
- Wanai (Mapoyo)
- Warao (Guarauno)
- Warekena (Walekhena)
- Wayuu (Guajiro, Goajiro)
- Wotjuja (Huotoha, Piaroa)
- Yabarana
- Yanomami (Guaica, Guaharibo)
- Yekuana (De´kuana, Maquiritare)
- Yukpa (Yucpa)
- White, non indigenous*
- Afrodescendent*
- Other.*

*Groups excluded from the datasets for the analysis of results of this paper.
