# Supplementary material for: The malaria burden of Amerindian groups of three Venezuelan states: a descriptive study based on programmatic data
Source: Malar J. 2021 Jun 26;20:285. doi: 10.1186/s12936-021-03819-7 (PMC8235908; doi:10.1186/s12936-021-03819-7)
Supplement: Supplementary file 5 — Additional file 5. Seasonal distribution of malaria cases diagnosed among Amerindian patients in Amazonas, Bolivar and Sucre between 2014 and 2018. [file 12936_2021_3819_MOESM5_ESM.docx]

Additional file 5: Seasonal distribution of malaria cases diagnosed among Amerindian patients in Amazonas, Bolivar and Sucre between 2014 and 2018


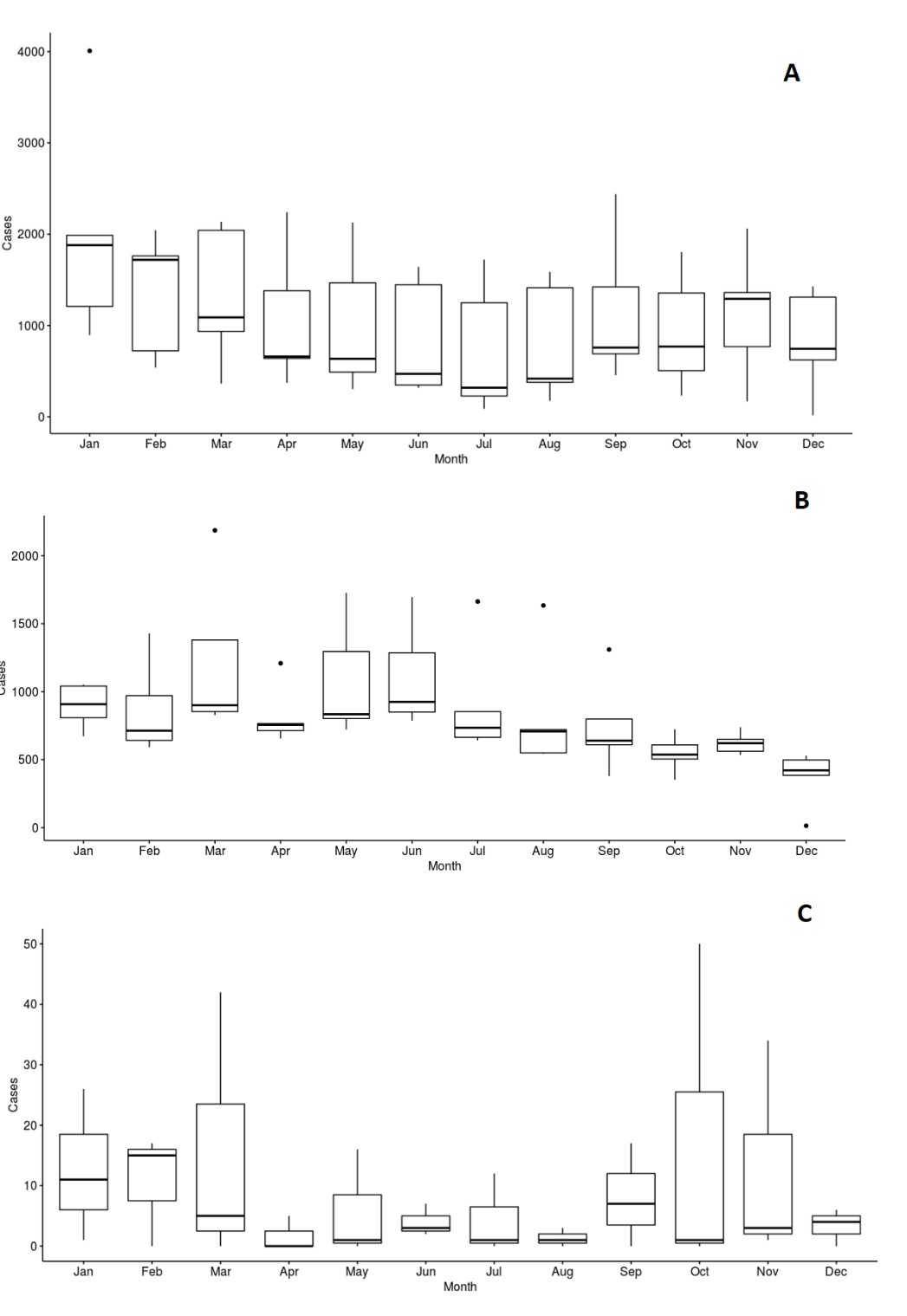


Kruskal Wallis test: A: Amazonas, *p=*0.57; B: Bolivar, *p*<0.01; C: Sucre, *p*=0.93
